# Impact of Circulating Tumor DNA–Based Detection of Molecular Residual Disease on the Conduct and Design of Clinical Trials for Solid Tumors

**DOI:** 10.1200/PO.21.00181

**Published:** 2022-03-09

**Authors:** Pashtoon M. Kasi, Gordon Fehringer, Hiroya Taniguchi, Naureen Starling, Yoshiaki Nakamura, Daisuke Kotani, Thomas Powles, Bob T. Li, Lajos Pusztai, Vasily N. Aushev, Ekaterina Kalashnikova, Shruti Sharma, Meenakshi Malhotra, Zachary P. Demko, Alexey Aleshin, Angel Rodriguez, Paul R. Billings, Axel Grothey, Julien Taieb, David Cunningham, Takayuki Yoshino, Scott Kopetz

**Affiliations:** ^1^ Department of Internal Medicine, University of Iowa Carver College of Medicine, Iowa City, IA; ^2^ Natera, Inc., Austin, TX; ^3^ Department of Gastroenterology and Gastrointestinal Oncology, National Cancer Center Hospital East, Chiba, Japan; ^4^ The Royal Marsden Hospital NHS Foundation Trust, London, United Kingdom; ^5^ Barts Cancer Institute, Queen Mary University of London ECMC, Barts Health, London, United Kingdom; ^6^ Memorial Sloan Kettering Cancer Center and Weill Cornell Medicine, New York, NY; ^7^ Yale Cancer Center, Yale School of Medicine, New Haven, CT; ^8^ West Cancer Center and Research Institute, Germantown, TN; ^9^ Georges Pompidou European Hospital, SIRIC-CARPEM, Université de Paris, Paris, France; ^10^ Department of Gastrointestinal Medical Oncology, The University of Texas MD Anderson Cancer Center, Houston, TX

## Abstract

Earlier detection of cancer recurrence using circulating tumor DNA (ctDNA) to detect molecular residual disease (MRD) has the potential to dramatically affect cancer management. We review evidence supporting the use of ctDNA as a biomarker for detection of MRD and highlight the potential impact that ctDNA testing could have on the conduct of clinical trials.

**METHODS:**

We searched the literature using MEDLINE (via PubMed) for articles from January 1, 2000, focusing on studies that assessed ctDNA as a predictor of cancer recurrence. Broadly focused searches on ctDNA and cancer were also performed to provide additional background information. www.clinialtrials.gov was searched to identify trials that incorporate ctDNA testing.

**RESULTS:**

Numerous studies across different cancer types indicate that ctDNA-based MRD detection predicts recurrence with high sensitivity and specificity, and with lead times that precede standard imaging by up to 12 months. Recently, ctDNA testing has started being used to enroll MRD-positive patients at high risk of recurrence into trials, promising gains in statistical power that allow clinical utility to be demonstrated with smaller cohorts. Trials where ctDNA testing based-MRD detection is used to stratify patients into low or high-risk categories for treatment assignment are also ongoing. In addition, there is increasing evidence supporting the use of ctDNA dynamics or clearance as a surrogate end point, which could significantly reduce trial duration.

**CONCLUSION:**

ctDNA-based trial enrichment across many cancers seems likely to become increasingly common for cost- and time-reduction benefits. Trial efficiency could also benefit from using ctDNA as a surrogate end point, leading to accelerated approval of new therapeutics. A clear demonstration of efficacy from trials that use ctDNA-based MRD detection to assign treatment could transform clinical practice.

## INTRODUCTION

The presence of extracellular DNA in blood, referred to as cell-free DNA (cfDNA), was first reported in 1948.^1^ The first reports of tumor-specific mutations in cfDNA were published in 1994, when *KRAS* and *NRAS* mutations were observed in patients with pancreatic cancer and acute myelogenous leukemia.^2,3^ Recent work indicates that for many cancers, there are genetic variants present in cfDNA that broadly overlap with variants found in tumor tissue.^4^ This tumor-derived fraction of cfDNA is commonly referred to as circulating tumor DNA (ctDNA). Over the past decade, the advent of next-generation sequencing (NGS) and other advances in methods for ctDNA detection contributed to a surge in research evaluating ctDNA as a cancer biomarker. The association of ctDNA with clinical variables has now been investigated in many cancers and it is well established that ctDNA levels are associated with stage, response to therapy, prognosis, and tumor burden.^4,5^

**CONTEXT**

**Key Objective**
Circulating tumor DNA (ctDNA) detection indicates the presence of molecular residual disease (MRD), identifying recurrence earlier than standard approaches. Key roles for ctDNA-based MRD detection in the design of clinical trials in adjuvant and neoadjuvant settings are examined.
**Knowledge Generated**
Globally, hundreds of clinical trials seek to show the benefit of therapeutic interventions. ctDNA-based MRD detection is increasingly being used to select patients at high risk for recurrence into clinical trials, as it can greatly reduce sample size and trial costs. Moreover, using ctDNA as a surrogate end point can result in substantial reductions in trial duration, expediting the introduction of new therapeutics into the clinic. The results from trials that investigate early therapeutic interventions after MRD detection could substantially affect clinical practice.
**Relevance**
ctDNA-based MRD detection could have a major impact on the conduct of clinical trials and ultimately on the management of disease in patients with cancer.

A significant new development in clinical cancer research is using ctDNA for detection of molecular residual disease (MRD) and molecular relapse. We use MRD (also referred to as molecular minimal residual disease) here to mean any molecular evidence of disease, typically when detected shortly after surgery or definitive treatment, whereas molecular relapse, treated here as a subset of MRD, is used to describe molecular evidence of disease found later, during treatment or surveillance. Numerous studies across different cancer types indicate that ctDNA-based MRD detection predicts recurrence with high sensitivity and specificity, preceding standard imaging by months.^6-12^

Reliable detection of MRD has substantial implications for clinical trial design. Identifying patients at high risk of recurrence through ctDNA testing can lead to substantial reductions in trial sample size, as enriching trials with patients likely to recur increases statistical power. Another potential role for ctDNA is as a surrogate end point for treatment response in settings where conventional response biomarkers are unavailable (eg, the adjuvant setting). This could provide an early indication of treatment efficacy relative to conventional measures such as progression-free survival and overall survival (OS). These gains in trial efficiency can reduce study costs leading to expedited approval of new therapies. ctDNA-based testing also provides the opportunity to conduct trials where MRD status guides treatment. These trials could determine whether MRD-positive patients benefit from early therapeutic interventions.

In this review, we elaborate on evidence supporting different approaches to using ctDNA in clinical trial design and discuss the utility of ctDNA-based MRD detection for increasing trial efficiency and guiding treatment across neoadjuvant and adjuvant settings.

## ctDNA DETECTION

ctDNA concentrations are often low (< 0.1% of cfDNA),^4,13^ particularly in early-stage cancers, and thus, methods with high analytic sensitivity are required for successful ctDNA detection. Detection methods include digital polymerase chain reaction,^14-16^ multiplex polymerase chain reaction–based NGS,^8^ and hybrid capture–based NGS.^17^ High costs of whole-genome and exome sequencing have discouraged efforts toward plasma-based detection of many types of somatic variants; however, whole-genome approaches for identifying genomic rearrangements in plasma have been used successfully in clinical research.^18^ Table 1 provides descriptions of ctDNA detection methods. Detailed reviews are provided elsewhere.^18,28^

**TABLE 1.**
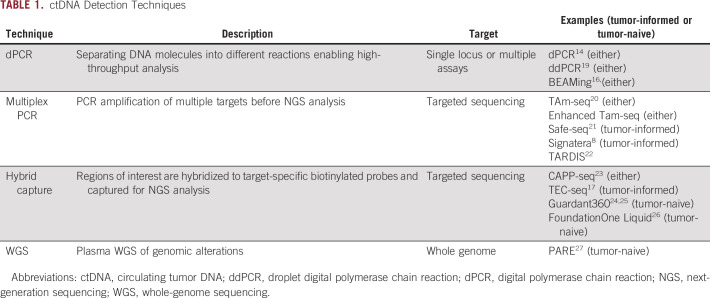
ctDNA Detection Techniques

Considerable attention has been given to broadly applicable assay strategies to enhance ctDNA detection accuracy. This includes assaying many variants instead of a single variant to increase the probability of finding detectable variants in plasma samples.^17,29^ A study that tracked multiple ctDNA variants in patients with stage I-III non–small-cell lung cancer following definitive treatment reported that a 94% detection rate with multiple markers dropped to 58% using the same platform with only a single marker.^29^ Recently, there has been a shift toward using personalized, tumor-informed approaches, where a patient's tumor biopsy results determine variants to be tracked in the plasma. These are suggested to have greater sensitivity than tumor-naive multigene panels, as the latter are reported to only detect an average of 2-5 variants per patient, despite using large panels (eg, 128 genes).^17,22,29^ Although multigene panels provide improved sensitivity relative to a single marker, a tumor-naive panel may not cover variants found in some patients. By contrast, prior knowledge from assessment of variants in tumor tissue allows tracking of a greater number of variants (often with relatively high frequency that permits more reliable detection), enhancing sensitivity.^22^ Knowledge of the tumor variant profile also ensures assays can be focused on variants present in plasma, whereas tumor-naive approaches assay many regions unlikely to contain a relevant variant, increasing false-positive results. False positives are a major clinical concern and a barrier to adoption if an intervention (eg, more imaging or systemic chemotherapy) is to follow a positive test.

Although tumor-informed approaches can reduce the proportion of false-positive tests, the probability of a false-positive result will still increase with the number of variants assayed. To combat this loss in specificity, some tumor-informed methods require at least two variants to be present in both tumor and plasma, substantially reducing false-positive results. Currently used tumor-informed approaches track different numbers of variants, ranging from 16 to as many as 115.^6,22^ It is as yet unclear how this difference in methodology, and other differences such as error models and assay design, influences specificity (and other accuracy measures).

Despite the benefits of using multiple variants and/or tumor-informed methods, costs of assaying multiple markers and availability of tumor tissue are potential barriers to using these approaches in some studies. Furthermore, the need to sequence tumor tissue for designing ctDNA assays can result in longer turnaround times for tumor-informed approaches (although this only affects the initial ctDNA test). Using presurgical material or designing assays promptly upon receipt of tumor material can ameliorate this concern.^30^ A summary of differences between tumor-informed and tumor-naive approaches is provided in Appendix Table A1.

Much of the focus of ctDNA testing involves detection of somatic mutations. However, aberrant changes in DNA methylation are widespread in tumor tissue and also reflected in plasma.^31^ The results from several studies indicate that DNA methylation holds promise for MRD detection.^32-35^ Current approaches generally examine methylation at a few cancer-related markers (eg, *SEPT9*, *BCAT1*, and *IKZF1*, in colorectal cancer [CRC]).^32-34^ Low costs and efficiency are cited as advantages of these assays.^35,36^ However, current results indicate that sensitivity for detecting MRD is lower than observed for mutation-based methods (eg, < 65% for either *SEPT9* or *BCAT1* and *IKZF1*^34,37^ *v* 80%-100% for somatic mutations^38-41^), although results for specificity are similar (80%-92%^34,37^ *v* 90%-100%^6,39,41^). These comparisons are hindered by small sample sizes and lack of uniformity in timing and frequency of ctDNA testing across studies, particularly in methylation studies. More research is needed to demonstrate the utility of methylation-based MRD testing.

Methylation approaches have also been used in tandem with testing of somatic mutations. A recent study, using a tumor-naive approach, reported that adding a predetermined methylation cancer signature to somatic genomic mutations increased sensitivity of detecting recurrence.^42^ More research is needed to further evaluate the combination of epigenomic and genomic markers in MRD testing. Of interest is whether the addition of genomic variants will improve the accuracy of tumor-naive approaches to levels comparable to tumor-informed approaches, and the impact on accuracy of adding methylation markers to current tumor-informed methods.

A significant challenge to maintaining specificity of ctDNA testing is confounding by clonal hematopoiesis of indeterminate potential (CHIP). CHIP mutations originate from hematopoietic progenitor cells.^43,44^ Recent studies have reported 14% of patients with early-stage lung cancer and 25% of patients with late-stage solid tumors harbor CHIP mutations.^45,46^ Because of the difference in methodology used to detect and define CHIP variants between these studies, a comparison of results does not permit inferences about CHIP mutation frequency by cancer type and stage. However, the high frequency of CHIP variants observed in both studies underlines how misclassifying CHIP variants as ctDNA variants may reduce specificity for MRD detection. Approaches to address this misclassification include sequencing paired peripheral blood mononuclear cells for in silico filtering of variants common to peripheral blood mononuclear cells and ctDNA, and using tumor-informed methods to identify clonal tumor variants.^8,47^

Many other factors can influence the accuracy of ctDNA detection, including tumor shedding, tumor location, weight, and recent surgery. We summarize these in Table 2.

**TABLE 2.**
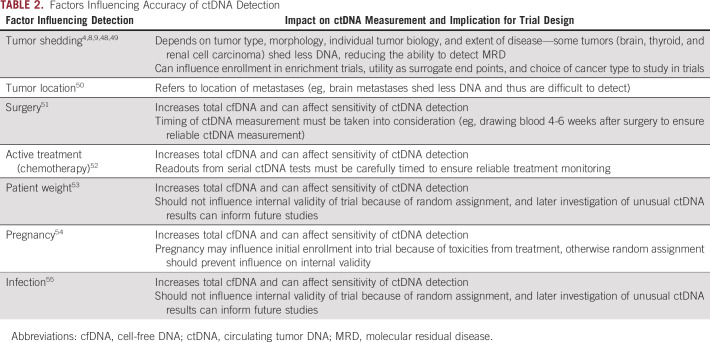
Factors Influencing Accuracy of ctDNA Detection

## MRD-BASED PATIENT ENROLLMENT IN CLINICAL TRIALS

There is substantial evidence that ctDNA-based MRD detection can stratify patients into high-risk and low-risk groups, which allows for more efficient trials through targeting high-risk patients for enrollment. Numerous retrospective studies across multiple cancer types have reported that ctDNA-based MRD detection is sensitive and specific for recurrence in both postoperative and serial testing scenarios (for some cancers, the latter can improve sensitivity of ctDNA testing relative to the postoperative setting, which we discuss further below.)

ctDNA-based MRD detection using serial testing predicted recurrence with 82%-100% sensitivity^6,38-41^ and 89%-100% specificity^6,39,41^ for CRC in the adjuvant setting, 79%-100% sensitivity and 100% specificity for breast cancer in the neoadjuvant or adjuvant setting,^7,56-58^ and 90% sensitivity and 88% specificity for pancreatic cancer in the adjuvant setting.^59^ Additional studies report ctDNA detected recurrence at 71% sensitivity and 100% specificity for esophageal cancer for a single ctDNA test performed after neoadjuvant therapy,^9^ and 94% sensitivity for non–small-cell lung cancer with a single test after local treatment (specificity was not reported).^29^ These studies used limited numbers of patients and require confirmation from large well-annotated cohorts. Still, because of the rapidly expanding body of evidence surrounding ctDNA-based MRD detection for CRC, a recently convened National Cancer Institute task force released a consensus statement concluding that the presence of ctDNA was strongly associated with a high risk of disease recurrence in CRC, with the results suggesting ctDNA was a robust marker for MRD.^60^ Currently, several ongoing trials are using ctDNA status to determine enrollment.

Restricting trial enrollment to those at high risk for recurrence is of clear benefit to patients. For example, patients with stage II CRC do not receive adjuvant chemotherapy, although 20% will experience recurrence.^61^ Enrollment on the basis of ctDNA-positivity ensures that only patients with a high probability of recurrence are included in the trial, whereas low-risk patients who are unlikely to benefit are spared from potential treatment-related side effects. Moreover, from a clinical perspective, most experts would agree on the value of offering systemic therapy for patients with low-risk stage II CRC with initial intent of observation and surveillance, given the 100% recurrence for ctDNA-positive patients noted across multiple studies.^6,62^

**FIG 1.**
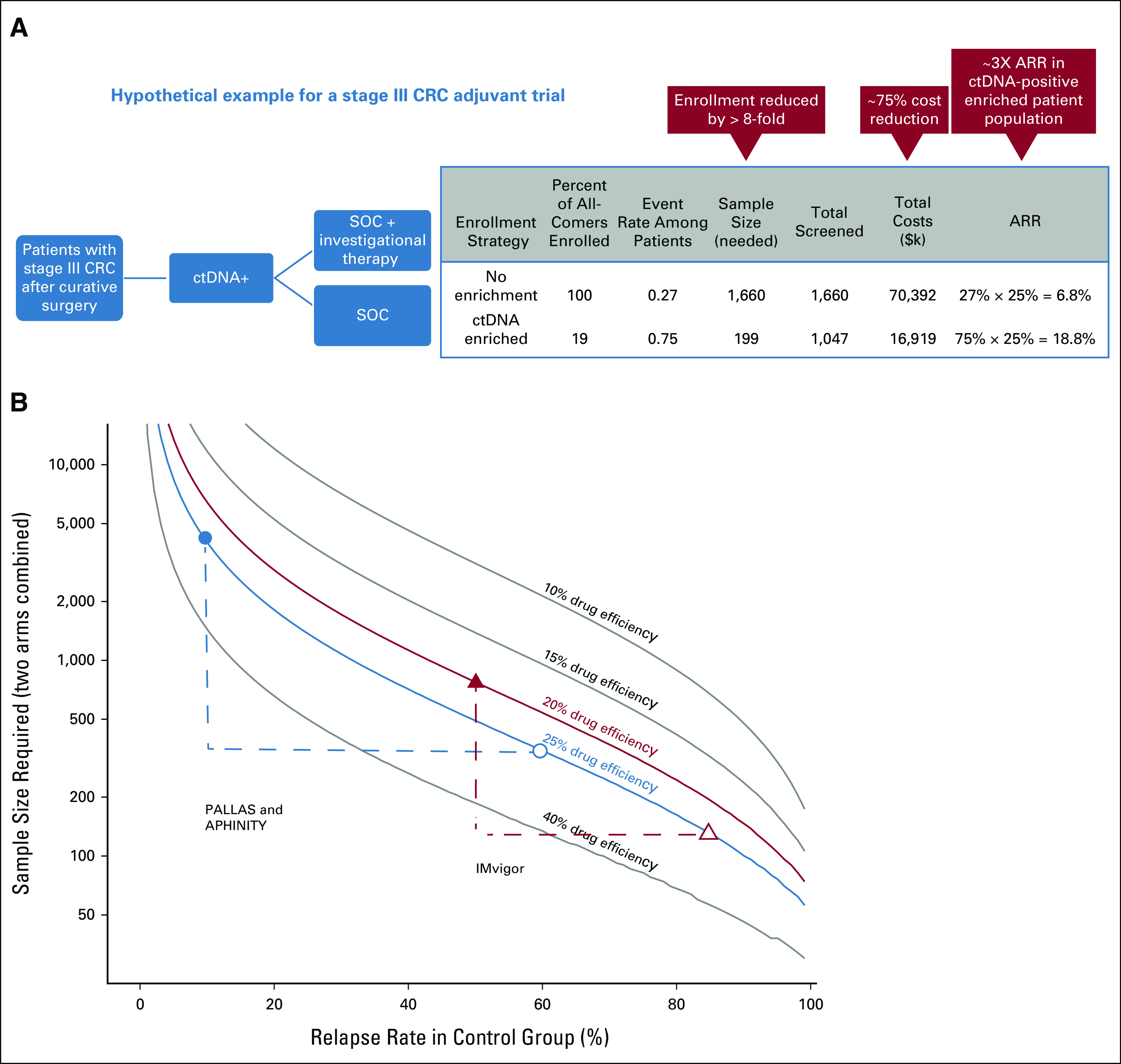
(A) Potential reduction in sample size and costs for stage III CRC trial through enrichment with ctDNA testing. In this scenario, an 8-fold reduction in enrollment size and a 75% reduction in per patient costs after accounting for treatment and ctDNA screening can be achieved (for sample size estimates, we assumed an event rate of 0.75 in ctDNA-positive patients in the control arm and a 0.25 relative risk reduction in the treatment arm.)^6^ (B) Decrease in sample size as related to relapse rate for disease in the control group at varying drug efficiencies. Enrollment through ctDNA testing has a dramatic impact on sample size, since the event rate is greatly increased if ctDNA-positive patients are selected. The plot shows potential decreases in sample size that could have been achieved for ongoing clinical trials had ctDNA testing been used for enrichment. The PALLAS and APHINITY studies were described in the text. We also show the sample size that could have been achieved through enrichment for the IMvigor010 trial (NCT02450331), a phase III randomized trial of adjuvant atezolizumab versus observation in patients with high-risk muscle-invasive bladder cancer, which originally enrolled 800 patients. Sample size estimates were obtained from the original study (the original sample size estimates for the PALLAS and APHINITY studies were smaller than the number eventually enrolled) and the same parameters from the original estimates were used to generate hypothetical sample sizes for the enriched studies with the exception that the observation arm event rate was changed to reflect recurrence in ctDNA-positive patients (0.6 for PALLAS and APHINITY on the basis of risk reduction estimates for patients receiving endocrine therapy^63^ and 0.85 for ctDNA-positive patients with urothelial carcinoma in the IMvigor010 observation arm^64^). Closed triangle and circle represent the original sample size estimates and open triangle and circle represent sample size estimates for enriched studies. ARR, absolute risk reduction; CRC, colorectal cancer; ctDNA, circulating tumor DNA; SOC, standard of care.

Improvement in clinical trial efficiency using ctDNA-based enrollment is highlighted in Figure 1A, where a scenario of clinical trial enrollment of ctDNA-positive stage III patients with CRC in the adjuvant setting is presented. We assume 19% of patients are ctDNA-positive, of which 75% will experience recurrence. The comparison group represents patients enrolled irrespective of ctDNA status, with a recurrence rate of 27%. Since the recurrence rate drives statistical power, smaller sample sizes are possible for ctDNA-positive cohorts. This results in a 6-fold reduction in enrollment and a 68% reduction in per patient costs after accounting for treatment and ctDNA screening.

Recent phase III adjuvant clinical trials that have enrolled thousands of patients further highlight the benefit of enriching trials with high-risk patients. For example, the PALLAS study enrolled 5,706 patients with early-stage hormone receptor–positive and human epidermal growth factor receptor 2–negative breast cancer in a trial with a planned 10 years of follow-up, to determine whether a CDK4/6 inhibitor added over a 2-year period to a minimum of 5-year standard-of-care (SOC) endocrine therapy improves disease-free survival (DFS). Similarly, the APHINITY study randomly assigned 4,805 patients with human epidermal growth factor receptor 2–positive breast cancer to investigate the addition of pertuzumab to chemotherapy plus trastuzumab. Both trials could have substantially reduced sample size and costs if reliable biomarkers to identify patients at high-risk for recurrence were available at enrollment (Fig 1B). Moreover, 5 years after the PALLAS study began enrolling patients, the second interim analysis did not demonstrate future trial efficacy. The APHINITY trial reported significantly lower invasive DFS with an absolute benefit at 6 years of 2.8%.^65^ However, the modest improvement in invasive DFS, a reported lack of benefit in node-negative patients, treatment toxicities, and the cost of running a lengthy trial with a large sample size underscore the importance of enrolling patients at high risk of recurrence into trials.^65,66^

Different ctDNA testing strategies may be used for trial enrichment. Often, a single postoperative test is used to immediately randomly assign patients into treatment arms. Other trials require alternative MRD testing strategies. Serial testing, often performed in the surveillance setting, can detect cancers that occur after definitive treatment. An added benefit is improvement in sensitivity for detecting recurrence for some cancers, as additional ctDNA tests, likely coupled with increased tumor shedding over time, will increase MRD detection. The IMvigor011 trial provides an example of this approach where ctDNA testing will be used to enroll patients up to 20 weeks after cystectomy (Table 3).

**TABLE 3.**
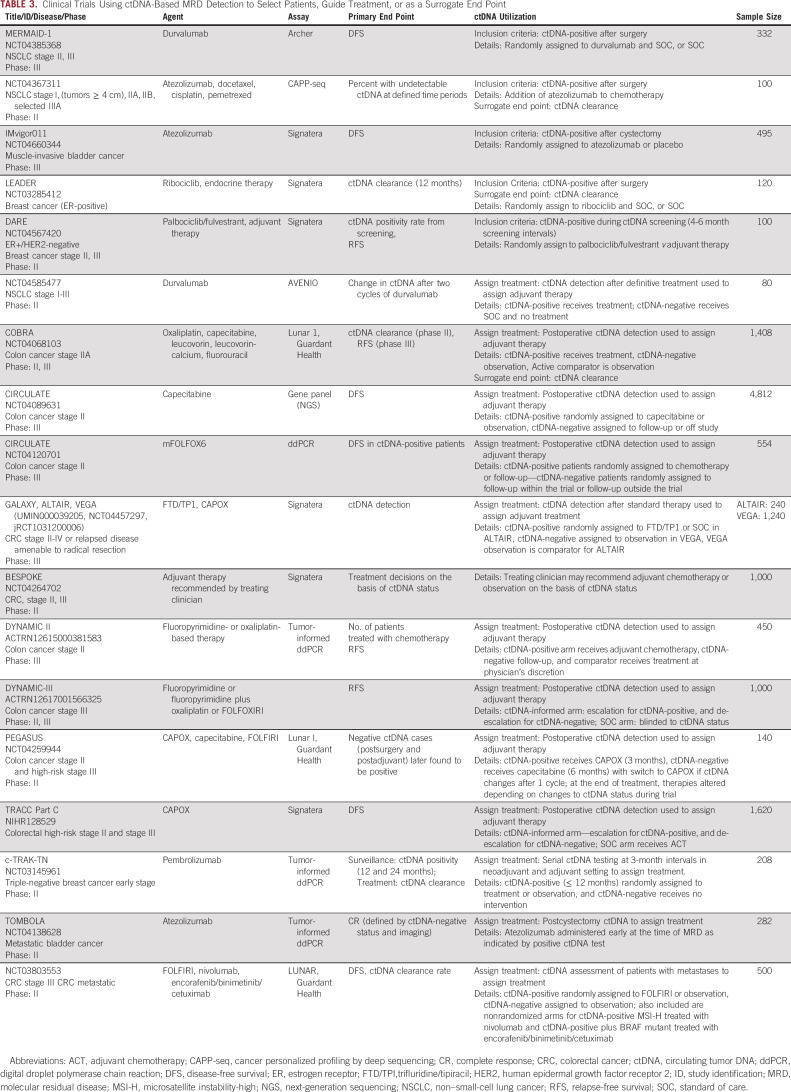
Clinical Trials Using ctDNA-Based MRD Detection to Select Patients, Guide Treatment, or as a Surrogate End Point

A paradigm shift is the extended serial testing that is possible with ctDNA. For example, up to 30% of patients with breast cancer relapse after definitive treatment, often years after their original diagnosis. In this setting, it is preferable to perform periodic ctDNA testing over an extended time period to assemble an enriched cohort.

At present, several registered trials are underway where ctDNA-positivity informs enrollment (Table 3, Appendix Table A2). Two of these, the MEDOCC-CrEATE CRC trial and the DARE breast cancer trial, conducted in the adjuvant and molecular recurrence settings, respectively, have published enrollment goals and size of the screened population needed to achieve these goals. Power calculations for MEDOCC-CrEATE indicated a sample size of 60 ctDNA-positive patients, which could be obtained from testing 1,320 patients, was sufficient for analysis of recurrence rates.^67^ The DARE trial estimated that 100 of 1,000 screened patients were needed to satisfy study power requirements for comparing recurrence across treatments. As screened populations are representative of sample size requirements for all-comers studies, these trials point to a 10- to 20-fold sample size reduction for ctDNA-based enrichment trials, consistent with scenarios described in Figure 1, and further support the using ctDNA-based enrichment studies to improve trial efficiency.

## MRD-BASED TREATMENT ASSIGNMENT IN CLINICAL TRIALS

A biomarker that detects recurrence before standard methods could have a major impact on outcome by identifying disease earlier when response to treatment is more likely. Studies that monitored ctDNA status during treatment or tested ctDNA postoperatively have shown that MRD detection in the adjuvant setting generally precedes SOC identified recurrence (Table 4). Stage I-III CRC studies reported ctDNA-detected median lead times of 1.8-11.5 months.^6,39,62,68^ For patients with breast cancer, lead times of 8.9-11 months were reported.^7,57^ Median lead times of 2.3-8.9 months were observed for lung, esophageal, gastric, and bladder cancer (Table 4).^8-11,29,70,71^ Some caution is warranted in interpreting the results as lead times are influenced by intervals between ctDNA testing and imaging, which vary across studies.

**TABLE 4.**
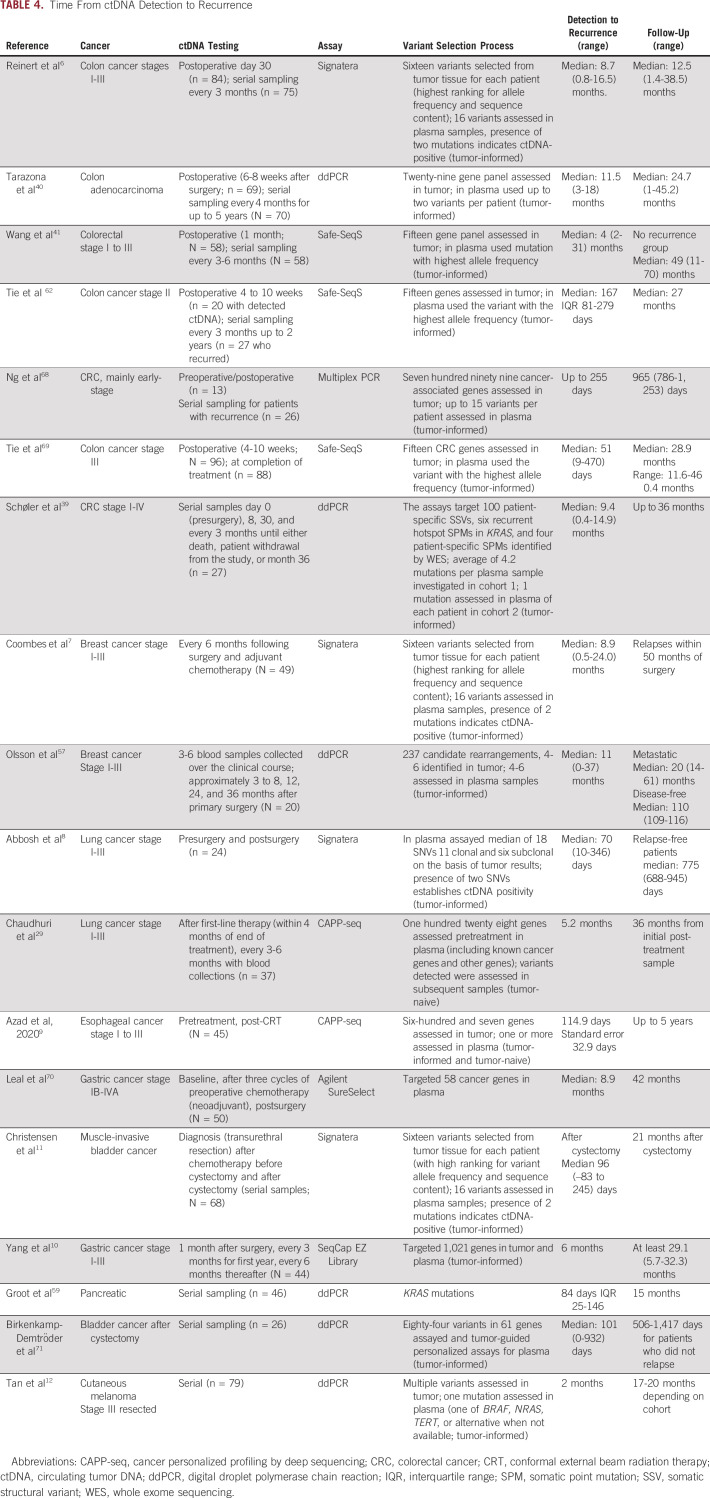
Time From ctDNA Detection to Recurrence

Currently, there are numerous randomized clinical trials testing the hypothesis that ctDNA-based MRD detection identifies patients at high risk of recurrence who may then benefit from early therapeutic interventions (Table 3, Appendix Table A2). Table 3 and Appendix Table A2 list trials where ctDNA status is used to assign adjuvant treatment.

**FIG 2.**
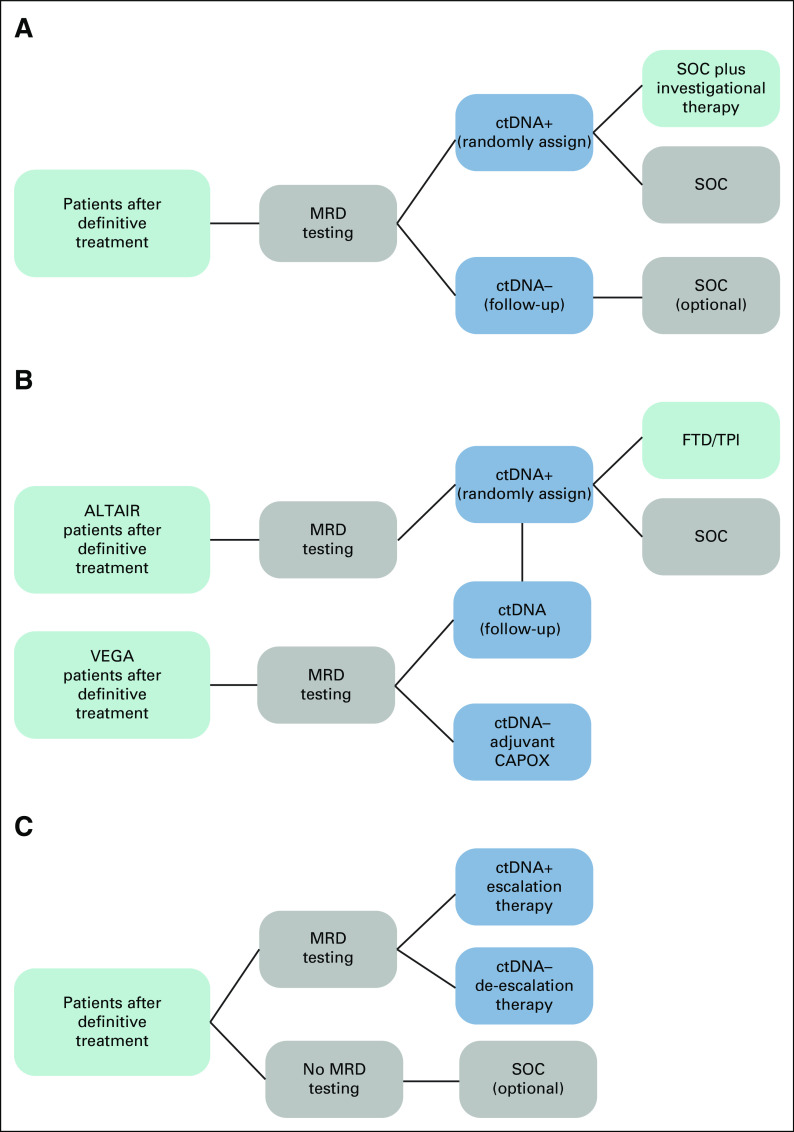
(A) Marker by treatment interaction design with MRD testing after definitive treatment. ctDNA-positive patients are randomly assigned to SOC plus investigational therapy versus SOC alone. ctDNA-negative patients are assigned to the follow-up group. Noninferiority component permits comparison of ctDNA-negative patients with ctDNA-positive patients to ensure these patients have outcomes that are no worse than treatment groups. (B) Marker by treatment interaction and noninferiority designs with MRD testing after definitive treatment (GALAXY, ALTAIR, and VEGA). ctDNA-positive patients from the GALAXY study are randomly assigned in the ALTAIR study to SOC plus investigational therapy versus SOC alone. ctDNA-negative patients from GALAXY are randomly assigned to CAPOX and follow-up. Noninferiority of follow-up versus CAPOX is investigated among ctDNA-negative patients. ctDNA-negative patients from VEGA who become ctDNA-positive can crossover to ALTAIR. (C) MRD testing after definitive treatment. The results of MRD testing are used to assign ctDNA-positive patients to escalation and ctDNA-negative patients to de-escalation therapy in Arm A. Arm B has no ctDNA testing and receives SOC. ctDNA, circulating tumor DNA; FTD/TPI, trifluridine/tipiracil; MRD, molecular residual disease; SOC, standard of care.

Several of these trials, including CIRCULATE, GALAXY with ALTAIR and VEGA, and c-TRAK-TN (Table 3), use a marker-by-treatment interaction design where ctDNA-positive patients are assigned to investigational therapy versus control (or escalation *v* de-escalation therapy), whereas ctDNA-negative patients receive SOC. This design permits comparison of the intervention on ctDNA-positive patients while ensuring that the ctDNA-negative group is noninferior to the intervention group (Fig 2A).

GALAXY and the related ALTAIR and VEGA studies provide a good illustration of such trial designs in the context of a large multicenter trial, encompassing both de-escalation (VEGA) and escalation (ALTAIR) trials and an observational study (GALAXY) that serves to screen patients for MRD, leading to their assignment to one of the two trials. These trials fall under the umbrella of CIRCULATE-IDEA (International Duration Evaluation of Adjuvant Chemotherapy Colon Cancer Prospective Pooled Analysis), a collaborative effort conducted by groups in Japan, the United States, Europe, and Australia to perform integrated analysis of data from ongoing randomized phase III studies.

The ALTAIR study is evaluating efficacy and safety of preemptive treatment with Trifluridine/tipiracil (FTD/TPI) compared with standard of care (SOC). Patients who test ctDNA-positive after undergoing curative resection in GALAXY will be recruited into ALTAIR and randomly assigned to treatment or control. VEGA tests noninferiority of observation versus adjuvant CAPOX. These trials incorporate a crossover component where VEGA participants who become ctDNA-positive can enter the ALTAIR trial (Fig 2B).

Trials such as DYNAMIC II, DYNAMIC III, and TRACC Part C use a marker-based strategy design framework. In these trials, patients are randomly assigned on the basis of MRD testing results, with ctDNA-positive patients assigned to treatment escalation and ctDNA-negative to de-escalation. An active comparator receives SOC in the absence of information regarding ctDNA status (Fig 2C).

The TRACC Part C study further illustrates the use of this design, examining whether postoperative ctDNA guided therapy (de-escalated chemotherapy for ctDNA-negative patients and SOC for ctDNA-positive patients) is noninferior to SOC chemotherapy in patients not tested for ctDNA. This highlights the possible role of ctDNA testing in reducing unnecessary SOC chemotherapy. Indeed, ctDNA testing may ultimately identify patients who are cured and do not require further therapy, a potential role supported by high DFS for ctDNA-negative patients in the adjuvant setting.^6,64,69^

Benefit from early detection is key to a successful intervention involving treatment assignment through disease monitoring. However, previous trials investigating intense monitoring versus SOC have not always shown benefit. Two randomized clinical trials in early-stage breast cancer that compared intense monitoring (serial chest X-rays and bone scans) to SOC failed to show improved survival.^72,73^ Likewise, serial carcinoembryonic antigen measurements and imaging (CRC^74^) and CA-125 assessments (ovarian cancer^75^) for monitoring patients following definitive treatment did not demonstrate a mortality benefit. Conversely, early salvage radiation on the basis of prostate-specific androgen testing is SOC for patients meeting specific risk-benefit criteria following definitive treatment.^76^ Furthermore, two recent randomized trials in prostate cancer demonstrated that early systemic therapy for rising prostate-specific androgen levels, in the absence of clinically detectable disease, can improve metastasis-free survival and has led to US Food and Drug Administration (FDA) approval of apalutamide and enzalutamide for this indication.^77,78^ MRD-directed therapy has also long been part of the treatment armamentarium in hematologic cancers. A recent example is the FDA-accelerated approval of blinatumomab for treatment of patients with a form of B-cell leukemia who had MRD after initial chemotherapy.^79^ This single-arm trial showed that those with a complete MRD response after blinatumomab had longer progression-free survival and OS durations. Trials that focus on ctDNA as an intervention are in the early stages, and conclusions regarding the clinical utility of ctDNA-based treatment assignment await their completion.

## SURROGATE END POINTS

Associations of ctDNA dynamics and clearance with response and survival outcomes are consistently reported across cancers in the neoadjuvant and adjuvant settings.^6,10-12,29,47,80-83^ These observations provide support for using ctDNA status as a surrogate that could act as an early indicator of clinical benefit, reducing trial length and accelerating approval of new therapeutics.

A role for surrogate end points in oncology trials is widely accepted, although debate exists regarding the employment of many existing surrogates.^84^ A surrogate end point validated against an established end point can provide insight into the benefit of new therapeutics, facilitating accelerated approval, although generally, a confirmatory trial, with potentially a large sample size, must be ongoing at time of approval. Pathologic complete response (pCR), discussed below, is a well-known surrogate end point. The FDA approved use of pCR for accelerated approval in the neoadjuvant setting for high-risk, early breast cancer in 2013.^85^

Initial data indicate that ctDNA holds promise as a surrogate end point. In the immuno-oncology setting, a minority of patients with solid tumors respond to immune checkpoint inhibitors (ICI), although treatment is known to have long-term benefits for responders. Early determination of response would enable patients who derive clinical benefit from immune checkpoint inhibitors to continue therapy while others could be spared from unnecessary toxicities.^86^ A retrospective analysis of the IMvigor010 trial found that patients with urothelial carcinoma undergoing adjuvant treatment with atezolizumab who cleared ctDNA had improved DFS (hazard ratio, 0.26 [95% CI, 0.12 to 0.56]) and OS (hazard ratio, 0.14 [95% CI, 0.03 to 0.59]) compared with patients who did not clear ctDNA. These results suggest that ctDNA testing provides an early readout that informs treatment decisions.^64^

A potential role for ctDNA testing is to be used as a complementary measure to pCR. A retrospective analysis of the I-SPY-2 clinical trial, which evaluated neoadjuvant treatment with investigational drugs in patients with high-risk breast cancer using pCR as an end point, provides support for using ctDNA testing in this role. The analysis found that ctDNA status was strongly associated with pCR, and lack of ctDNA clearance was a predictor of poor response and metastatic recurrence. Importantly, ctDNA clearance was associated with improved survival in patients who did not achieve pCR, indicating ctDNA testing might provide information regarding outcome in clinical trials beyond that of pCR.^83^ Interestingly, data from this trial also indicated ctDNA could serve as a complement to MRI functional tumor volume as a predictor of treatment response.^87^

Further evidence is needed before ctDNA dynamics or clearance can be approved as surrogate end points in trials. Approval by appropriate regulatory agencies depends on accumulation of evidence from observational studies and clinical trials. Meta-analyses of clinical trials will be key in determining whether ctDNA dynamics or clearance robustly predicts treatment effect on the true end point.^88^

Currently, meta-analyses investigating ctDNA clearance as an end point in the adjuvant setting are planned for CRC. In addition, ongoing trials are collecting ctDNA as secondary measures permitting further evaluation across cancer types and treatment settings. Analysis and interpretation of these data has challenges, because of heterogeneity in study design, ctDNA assays, measurement and metrics, and timing of ctDNA samples. Preliminary work from ctMoniTR, a collaboration of private, government, and academic institutions with the aim of harmonizing data from clinical studies using ctDNA-based treatment response monitoring, indicates that trends in individual trials can be replicated in aggregate.^88^ Agreements on standardization around key aspects of trial design, such as ctDNA collection time points, would further streamline analyses and facilitate interpretation of results.^60^

## CHALLENGES AND LIMITATIONS

As highlighted, alongside evidence presented for using ctDNA for MRD detection in various malignancies, the clinical question, assay timing, biology of shedding for different cancer types, and number of time points being collected cannot be ignored. Furthermore, reliable knowledge of recurrence rates of ctDNA-negative patients by cancer type is needed to allow for appropriate and ethical de-escalation or discontinuation of therapy. The merits of ctDNA testing would have to be weighed with the risks and long-term quality-of-life outcomes. Additionally, a question that often arises is whether ctDNA-positive patients are treatable or have occult metastatic disease that may not be cured with adjuvant therapy. The latter two concerns underscore the need to consider possible harms from molecular monitoring and early intervention, particularly if clinical utility is less than what is currently anticipated. Costs of ctDNA testing may represent added out-of-pocket expense for patients. False-positive results can lead to additional testing, mental stress for patients, and exposure to toxic and unnecessary therapy. Even true-positive results can lead to deterioration of quality of life because of drug-induced adverse effects at a stage when the cancer is asymptomatic. Early systemic therapy for patients who relapse may also run the risk that an effective treatment option will no longer be available when the cancer becomes symptomatic. These concerns can be settled through well-conducted clinical trials.

In conclusion, evidence pointing to ctDNA as a biomarker that can predict cancer recurrence continues to accumulate. ctDNA-based MRD detection is now being incorporated into the design of clinical trials. It seems likely that, for many cancers, ctDNA-based trial enrichment will become increasingly common because of cost-reduction benefits. Trial efficiency could also benefit from using ctDNA as a surrogate end point, leading to accelerated approval of new therapeutics. Further research, however, is needed to validate ctDNA dynamics or clearance as end points. In the next few years, trials using ctDNA-based MRD detection to identify patients who may benefit from early therapeutic interventions will release results. A clear demonstration of efficacy of ctDNA-based MRD detection would transform clinical practice. The importance of enrolling patients into ongoing trials incorporating ctDNA as an integral or exploratory marker cannot be overemphasized.

## Figures and Tables

**TABLE A1.**
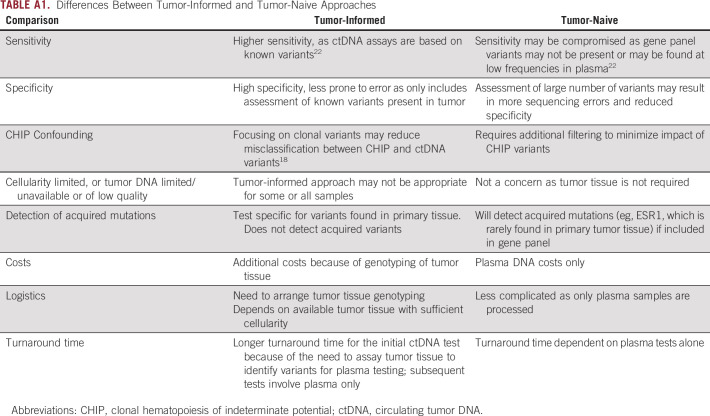
Differences Between Tumor-Informed and Tumor-Naive Approaches

**TABLE A2.**
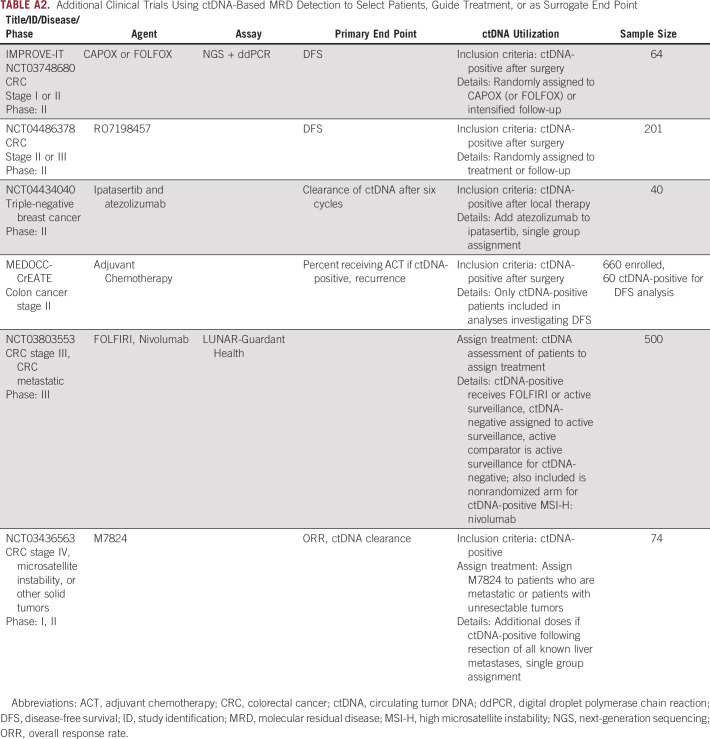
Additional Clinical Trials Using ctDNA-Based MRD Detection to Select Patients, Guide Treatment, or as Surrogate End Point
